# Development of a Microfluidic-Based Optical Sensing Device for Label-Free Detection of Circulating Tumor Cells (CTCs) Through Their Lactic Acid Metabolism

**DOI:** 10.3390/s150306789

**Published:** 2015-03-19

**Authors:** Tzu-Keng Chiu, Kin-Fong Lei, Chia-Hsun Hsieh, Hung-Bo Hsiao, Hung-Ming Wang, Min-Hsien Wu

**Affiliations:** 1 Department of Chemical and Materials Engineering, Chang Gung University, Taoyuan 33302, Taiwan; E-Mails: b74225@hotmail.com (T.-K.C.); wisdom5000@gmail.com (C.-H.H.); 2 Graduate Institute of Medical Mechatronics, Chang Gung University, Taoyuan 33302, Taiwan; E-Mail: kflei@mail.cgu.edu.tw; 3 Department of Mechanical Engineering, Chang Gung University, Taoyuan 33302, Taiwan; 4 Division of Hematology-Oncology, Department of Internal Medicine, Chang Gung Memorial Hospital at Linkuo, Taoyuan 33302, Taiwan; 5 Graduate Institute of Biochemical and Biomedical Engineering, Chang Gung University, Taoyuan 33302, Taiwan; E-Mail: harber0815@gmail.com; 6 Division of Hematology-Oncology, Department of Internal Medicine, Chang Gung Memorial Hospital, Chang Gung University, Taoyuan 33302, Taiwan; E-Mail: whm526@gmail.com

**Keywords:** microfluidic technology, optical sensing, circulating tumor cells (CTCs), cancer cells, lactic acid

## Abstract

This study reports a microfluidic-based optical sensing device for label-free detection of circulating tumor cells (CTCs), a rare cell species in blood circulation. Based on the metabolic features of cancer cells, live CTCs can be quantified indirectly through their lactic acid production. Compared with the conventional schemes for CTC detection, this label-free approach could prevent the biological bias due to the heterogeneity of the surface antigens on cancer cells. In this study, a microfluidic device was proposed to generate uniform water-in-oil cell-encapsulating micro-droplets, followed by the fluorescence-based optical detection of lactic acid produced within the micro-droplets. To test its feasibility to quantify cancer cells, experiments were carried out. Results showed that the detection signals were proportional to the number of cancer cells within the micro-droplets, whereas such signals were insensitive to the existence and number of leukocytes within. To further demonstrate its feasibility for cancer cell detection, the cancer cells with known cell number in a cell suspension was detected based on the method. Results revealed that there was no significant difference between the detected number and the real number of cancer cells. As a whole, the proposed method opens up a new route to detect live CTCs in a label-free manner.

## 1. Introduction

Cancer metastasis is a primary cause of cancer-derived death [1]. Circulating tumor cells (CTCs) are a rare cell species present in the peripheral blood that has been documented since 1869 [2]. It is well recognized that the presence of CTCs in the blood circulation is thought to be mainly responsible for cancer progression or relapse [2]. The detection of CTCs in blood holds great promise to: (1) evaluate cancer stage [3], (2) predict the prognosis of cancer survival [4,5,6], (3) monitor disease recurrence or progression [7], and (4) assess cancer patients’ responses to particular therapeutic regimens [8]. Compared with the analysis of solid tumor tissues through an invasive process, the key clinical advantage of CTC detection is that it is relatively safe and easy to do through a blood sample, and can be performed at any time point during the cancer care process. Nevertheless, CTCs are very rare, with an approximate concentration of 1 CTC per 10^5^–10^7^ mononuclear blood cells [9], making them technically demanding to detect. 

The current methods for CTC detection consist of two steps, the CTC isolation process and the identification process. With the recent advances in cell isolation and separation techniques, the detection of CTCs with a high degree of specificity and sensitivity has been realized through various conventional strategies, which can be broadly categorized into physical and biochemical schemes [10]. Overall, the physical methods (e.g., size [11], electrical properties [12], or deformability [13] based methods) for CTC detection are normally easy to perform and label-free, but are not as specific and sensitive as the biochemical counterparts. Alternatively, biochemical techniques [14] mainly make use of the coupling of magnetic microbeads with CTC surface antigen-specific antibodies to recognize, and then capture the CTCs. In such immuno-magnetic microbead-based cell isolation schemes, the targeted surface antigens on CTCs are either tumor-specific markers, or epithelial-specific markers. For the latter, the most commonly-used biomarkers are epithelial cell adhesion molecule (EpCAM), and cytokeratins (CKs) [15], which are expressed by cancer cells of epithelial origin, and are normally absent in normal blood cells. The magnetic microbead-bound CTCs are then separated from the leukocyte background through an applied magnetic field. The cell isolation based on this strategy is usually referred to as positive selection of target cells, which is the predominant method of choice in current CTC detection methods. 

Although the above CTC detection methods have been proven promising to isolate or detect CTCs, there are some important biological issues that need to be further considered. Briefly, most of immune-magnetic-microbead-based CTC detection schemes rely highly on the use of the two epithelial markers to identify or capture CTCs. Reports in the literature, however, have revealed that EpCAM or CKs are not expressed in all tumors (e.g., sarcoma or melanoma), and therefore some kinds of CTCs cannot be targeted via these positive selection-based schemes [16]. In addition, CTCs, particularly those of a highly invasive and metastatic nature, might undergo a so-called epithelial-to-mesenchymal transition (EMT) [17], by which the epithelial tumor cells might alter their morphology, reduce cell adhesion ability, down-regulate the expression of EpCAM and CKs [16], and become motile cells for the migration to the distant metastatic sites [18]. Due to the phenotypic heterogeneity, the risk of false negative CTC detection could occur when epithelial biomarker targeted immuno-based methods are adopted. Conversely, it was also reported that non-epithelial cells can express the epithelial biomarkers, and again, this abnormality could lead to false positive results [19]. As a whole, the positive selection of CTCs using immuno-based methods targeting epithelial biomarkers can possibly lead to biased outcomes, by which, more importantly, clinically-meaningful CTCs associated with metastatic diseases might be missed. 

To address these issues, some studies have proposed some negative selection-based strategies for CTC detection [20], by which, conversely, the leukocytes in a blood sample are targeted and then removed through an immuno-magnetic microbeads-based approach leaving behind the all possible CTCs in the sample. To the best of our knowledge, however, most of these methods normally suffer from low purity of isolated CTCs [21], which could make the subsequent CTCs detection work technically-demanding and time-consuming. In addition to the abovementioned technical issues, overall, the biochemical approaches utilize antibodies specific to the surface antigens on CTCs for capturing and detecting CTCs. Commercial antibodies are normally expensive and, thus, this makes CTC detection assays costly. This economical issue can hinder the widespread application of the above assays for CTC detection.

In order to detect the all possible CTCs in a sample without the mentioned biological bias and particularly in a low cost manner, we propose to detect such cells indirectly through their metabolic feature of lactic acid production. It is well recognized that most cancer cells mainly produce energy through glycolysis and the subsequent lactic acid fermentation in the cytosol, rather than by glycolysis followed by oxidation of pyruvate in mitochondria like most biological cells [22,23]. In cancer cell cultures, the lactic acid produced by cancer cells is commonly released to the culture medium. By measuring the lactic acid in the culture medium, the live cancer cells could be indirectly detected in a quantifiable manner. In general, the quantification and detection of lactic acid can be achieved in a simple, precise, and particularly low cost manner. As described earlier, however, CTCs are scarce in a blood sample, and such rarity could make the lactic acid produced by them difficult to quantify using the conventional assays for lactic acid measurement. 

To tackle this technical hurdle, a blood cell suspension with an added fluorescence-based lactate reagent was uniformly segmented into multiple micro-droplets in this study. Due to the tiny volume of a micro-droplet (e.g., 0.004 μL), the resulting lactic acid concentration in the micro-droplets is technically feasible to measure. With the recent progress in microfluidic technology, microfluidic devices capable of continuously generating micro-droplets have been actively developed for various applications [24,25,26], which have also been well reviewed elsewhere [27,28]. In this study, a microfluidic device with such a function was designed and fabricated. In its operation, a quantitative link between the flow rates of oil and suspension, and the resultant size (diameter) of water-in-oil micro-droplets was established. The results showed that the proposed microfluidic device was able to generate cell-containing micro-droplets in a size-tunable, size-uniform, and cell-friendly manner. To test the feasibility of using the proposed approach to quantify live cancer cells, the fluorescence signals of micro-droplets generated from the waste cell culture mediums with different cell numbers of leukocytes, and cultured human oral cancer (OEC-M1) cells were detected and compared. Results showed that detection signals were proportional to the number of cancer cells cultured in such a medium, whereas such signals were insensitive to the existence and number of leukocytes in the medium. To further demonstrate its feasibility for cancer cell detection, known numbers of cancer cells were detected in a prepared cell suspension based on the proposed method. The results revealed that there was no significant difference between the number detected and the real number of cancer cells. Overall, this study has proposed a microfluidic-based optical sensing device to detect live CTCs in an effective and economical manner.

## 2. Experimental Section

### 2.1. Design of Microfluidic Device 

**Figure 1:**
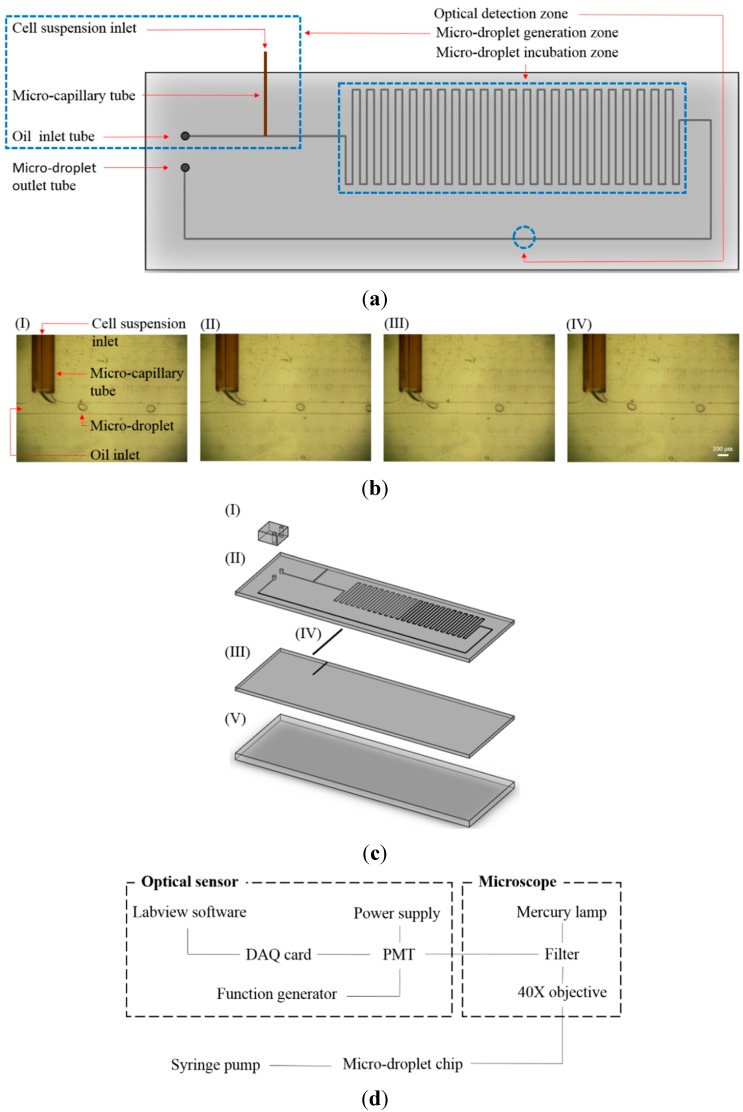

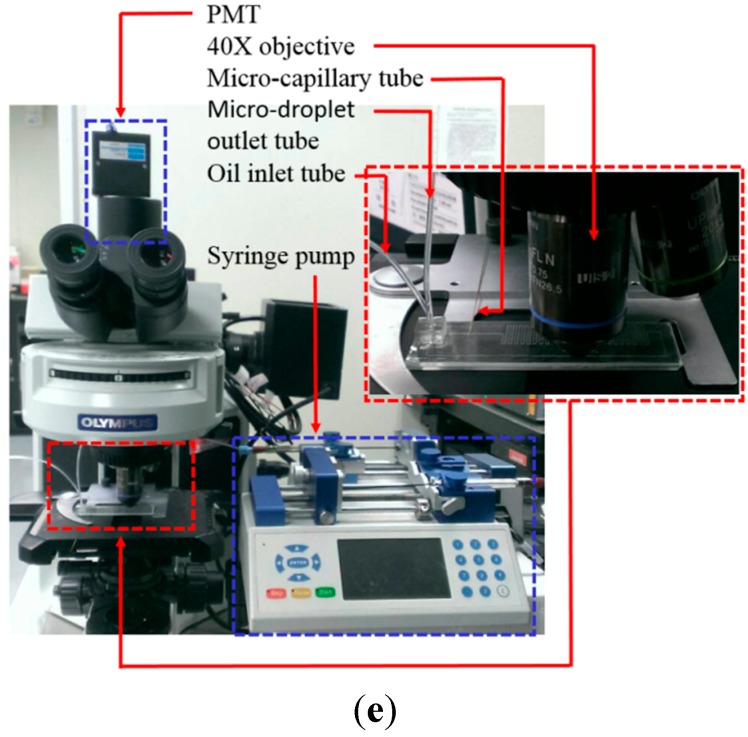
(**a**) Schematic illustration of the microfluidic device (Top-view layout); (**b**) The photographs of the continuous micro-droplet generation process ((I)-(IV)); (**c**) Schematic illustration of the assembly of the microfluidic device ((I)-(III): microfabricated PDMS layers, (IV): micro-capillary tube, (V): glass layer); (**d**) schematic illustration; and (**e**) photograph of overall experimental setup.

The proposed microfluidic device mainly integrates the functions of continuous cell-containing micro-droplet generation and the subsequent fluorescence-based optical sensing of lactic acid. Its layout is schematically illustrated in Figure 1a. Briefly, the microfluidic device consists of micro-droplet generation, micro-droplet incubation, and optical detection zones. In the micro-droplet generation zone, a cell suspension supplemented with a fluorescence-based lactate reagent (Lactate Fluorometric Assay Kit, BioVision, Milpitas, CA, USA) was continuously delivered to the micro-channel for oil (mineral oil) flow (L: 10 mm, W: 200 μm, H: 100 μm) through a micro-capillary tube (OD: 375 μm and ID: 50 μm). By this simple process, cell suspension micro-droplets were generated in a continuous manner (Figure 1b). The water-in-oil micro-droplets formed were subsequently delivered to the micro-droplet incubation zone, in which the micro-channel (L: 620 mm, W: 200 μm, H: 100 μm) was designed in meandering manner to accommodate more cell-containing micro-droplets. After the incubation zone was fully filled with micro-droplets, the input flows (namely the cell suspension and oil flows) were stopped so as to keep the micro-droplets in the incubation zone in a static manner for up to 3 h. This process not only allows the cancer cells in the micro-droplets to produce lactic acid for the assay but also allows the development of fluorescence for the subsequent optical sensing step. After a static incubation, the micro-droplets were delivered to the micro-channel (L: 66 mm, W: 200 μm, H: 100 μm) parallel to the micro-droplet generation, and incubation zones for the quantification of lactic acid through the fluorescence-based optical sensing (Figure 1a). After that, the micro-droplets were continuously removed from the microfluidic system via an outlet tube. The assembly of the microfluidic device is schematically illustrated in Figure 1c. Briefly, it consists of three polydimethylsiloxane (PDMS) layers (Layer I to III), one micro-capillary tube (IV), and one glass layer (Layer V). Structurally, the PDMS Layer I containing two vertical holes (D: 1 mm, H: 3 mm) was designed to accommodate the oil inlet and micro-droplet outlet tubes. The micro-channels for oil, and micro-droplet flows as well as the micro-channel for the insertion of micro-capillary tube were fabricated on the PDMS Layer II. PDMS Layer III is comprised of one micro-channel (L: 8 mm, W: 250 μm, H: 150 μm) for the physical insertion of micro-capillary tube. In addition, a transparent glass plate (thickness: 1 mm) was used as a supportive material at the bottom of the microfluidic device.

### 2.2. Fabrication and Experimental Setup

The overall fabrication processes was based on a computer numerical control (CNC) milling process for mold making, PDMS (Sylgard^®^ 184, Dow Corning, Midland, MI, USA) replica molding, and bonding processes as described previously [29]. For the PDMS Layer II and III, two polymethylmethacrylate (PMMA) molds with the desired microstructures were created using a CNC miller (EGX-400, Roland Inc., Irvine, CA, USA) with a 0.4 mm drill bit (rotational speed: 26,000 rpm). In the following replica molding process, PDMS was prepared by thoroughly mixing the PDMS pre-polymer with a curing agent in a ratio of 10:1 by weight. The polymer was then deaerated under vacuum to remove any air bubbles generated during mixing. Then the mixture was poured onto the two fabricated PMMA molds and cured at 70 °C for 1 h. The cured PDMS Layer II and III were then obtained after a de-molding process. For the PDMS Layer I, two vertical holes were simply created by drilling through a PDMS plate using an 18 G stainless needle. In this study, the three PDMS layers (Layer I to III), and the glass plate (Layer V) were permanently bonded by a plasma oxidation treatment which is followed by the insertion of the micro-capillary tube (IV) into the designed micro-channel.

In operation, the microfluidic system was placed on a tunable microscope stage. The inlet tubes for oil and cell suspension were separately connected to two syringe pumps (Fusion 200, Chemyx Inc., Stafford, TX, USA) for driving the two flows with different flow rates. For the fluorescence detection, a mercury lamp associated with a specific optical filter was used to provide an excitation light with wavelength of 530 nm. In this work, the emitted fluorescence from each cell-containing micro-droplet was detected using a photomultiplier tube (PMT) module (H7828, Hamamatsu, Japan) and its associated power supply, and function generator. The optical signal received was then digitized and acquired by a data acquisition (DAQ) card (PCI 6221, National Instrument, Austin, TX, USA) module via a personal computer (running the software LabVIEW). The schematic illustration and photograph of the experimental setup are shown in Figure 1d,e, respectively.

### 2.3. Evaluation of Size and Uniformity of Micro-Droplets

In this study, the size of the generated micro-droplets is mainly determined by the flow rates of the cell suspension, and oil separately driven by two syringe pumps. In order to investigate the quantitative relationship between the operating conditions and the size of the resultant micro-droplets, the following experiments were carried out. Briefly, the oil flow rates were set at three different levels (250, 500 and 750 µL·h^−1^). At a given oil flow rate, the flow rate of cell suspension was set from 60 to 140 µL·h^−1^. The micro-droplets generated under these investigated conditions were collected, and images of the micro-droplets (about 100 micro-droplets/image) were then captured using a digital camera-coupled microscope. The sizes of the micro-droplets were then measured using an image analysis software (SimplePCI version 5.2.1, Compix Inc., Cranberry Township, PA, USA) to determine the diameter of micro-droplets, based on our previous study [30]. The mean value of micro-droplet diameters, and the coefficient of variation (C.V.) (the ratio of the standard deviation to the mean value of the diameter) were then obtained from this data. 

### 2.4. Evaluation of Cell Viability 

In this work, the micro-droplets were generated to encapsulate cells both for a short period of incubation, and lactic acid measurement. For this application, a cell-friendly process is required to maintain high cell viability. The following experimental results were presented to evaluate the impact of the proposed micro-droplet generation process on cell viability. Briefly, leukocyte, and OEC-M1 cell suspensions (cell density: 2 × 10^6^ cells·mL^−1^) were separately loaded into the proposed microfluidic device. In the testing, the flow rates of cell suspension and oil were set at 110 and 750 µL·h^−1^, respectively (estimated diameter of micro-droplet: 225 µm). After the micro-droplet generation process, the viability of the encapsulated leukocytes, and OEC-M1 cells in the micro-droplets was then evaluated using a fluorescent dye kit (LIVE/DEAD^®^ Viability/Cytotoxicity Kit L-3224, Molecular Probes, Grand Island, NY, USA) [30]. All assays were performed according to the manufacturer’s instructions. Briefly, 200 μL of the dye reagent containing 1 μM calcein AM and 2 μM ethidium homodimer-1 was loaded onto the collected cell-containing micro-droplets. After a 20 min incubation period, the images of live (green) and dead (red) cells were captured using a digital camera-coupled fluorescence microscope. Cell viability was then estimated by counting the live (green) and dead (red) cells using an image acquisition software (SimplePCI version 5.2.1) [30]. In this study, the observation of the cell viability of the leukocytes, and OEC-M1 cells after 3 h incubation at room temperature was also conducted.

### 2.5. Quantification of the Live Cancer Cells through Fluorescence-Based Optical Detection of Lactic Acid

In this study, the live cancer cells in the micro-droplets were quantified indirectly through their lactic acid production. To achieve this, a commercially-available fluorescent dye was supplemented in the tested cell suspension. During operation, the cell-containing micro-droplets were kept in the incubation zone of themicrofluidic device (Figure 1a) in a static manner for a short period of time, allowing the production of lactic acid by cancer cells, and the development of fluorescence. In order to find out the incubation time after which the development of fluorescence is adequate for the following optical sensing, an experiment was carried out. Briefly, 2000 μL of lactic acid solution (0.6 nmol) was mixed with 1.25 μL of fluorescent reagent in a 96-well microplate according to the manufacturer’s instructions. The fluorescence at 590 nm was measured periodically using a microplate reader (Sunrise, Tecan Ltd, Taipei, Taiwan) for up to 22 h. Based on this, a quantitative link between the incubation time and the fluorescent intensity developed was established.

In order to test the feasibility of using such approach for cancer cell detection, the following experiments were carried out. Briefly, cell suspensions of leukocytes, and OEC-M1 cells with different cell densities (2.5 × 10^5^, 5 × 10^5^, 1 × 10^6^, 2 × 10^6^, 4 × 10^6^ cells·mL^−1^; the corresponding cell numbers in each 0.004 μL micro-droplets were 1, 2, 4, 8, 16 cells·droplet^−1^, respectively) were loaded into the wells of 96-well microplate for a 3 h static incubation. After incubation, the cell suspensions were loaded into the microfluidic device followed by the micro-droplet generation process described previously. In this study, the flow rates of oil and cell suspension were set at 750 and 110 µL·h^−1^, respectively, to generate micro-droplets with an estimated diameter of 225 µm (volume: 0.004 μL). The detection signals of each micro-droplet were measured based on the established experimental setup (Figure 1d,e) as described earlier. The quantitative relationship between the detection signals and the corresponding cell number in each micro-droplet was then established. After the fundamental investigations, a cell suspension containing a known number of cancer cells (OEC-M1 cells: 10 cells; volume: 10 µL) was loaded into the proposed microfluidic device. The flow rates of oil and cell suspension were set at 750 and 110 µL·h^−1^, respectively to generate micro-droplets with an estimated diameter of 225 µm (volume: 0.004 μL). The cancer cells were then experimentally quantified based on the established method described earlier. 

### 2.6. Statistical Analysis

Data from at least three separate experiments were analyzed and presented as the mean ± the standard deviation. For a given experiment, each condition was tested in triplicate. One-way ANOVA analysis with a statistical significance level of 0.05 was used to examine the effects of cell number on the detection signals measured. The Tukey Honestly Significant Difference (HSD) *post hoc* test was used to compare the detection signal differences between two tested conditions with different cell number when the null hypothesis of ANOVA analysis was rejected.

## 3. Results and Discussion

### 3.1. The Application of the Proposed Microfluidic Device for Micro-Droplet Generation and Microencapsulation of Cells

Emulsification-based methods are conventionally used for producing cell-encapsulating micro-droplets, or microbeads. In the process, a cell suspension and a biomaterial mixture are mechanically mixed to generate tiny aqueous cell-containing droplets within an oil phase. With the recent advances in microfluidic technology, this has paved a new route to generate cell-encapsulating micro-droplets with superior properties in comparison with those based on conventional methods. It is accepted that the key technical features of using microfluidic technology for the generation of cell-containing micro-droplets are its inherent ability to produce such micro-droplets of uniform size [24,31,32], and with a high level of sterility due to the operation in a confined and continuous microfluidic system. These characteristic traits are found particularly useful in subsequent biomedical applications, or studies [28]. Microfluidic devices with different designs or working principles (e.g., T-junction [33], Y-junction [34], Cross junction [35], Microfluidic Flow Focusing Devices (MFFD) [36]) have been actively proposed to generate cell-containing micro-droplets for various applications (e.g., single-cell analysis [26], drug screening [37], enzyme analysis [38], genetic analysis [39]). Nevertheless, the current microfluidic-based methods for cell-containing micro-droplet generation are normally complicated in terms of device fabrication, and operation. To tackle these technical issues, this study simply used a T-shaped microchannel to continuously generate cell-containing aqueous micro-droplets in an oil stream, as shown in Figure 1b. In the process, an aqueous cell suspension was continuously delivered into an oil flow. Due to the insolubility of these two materials, the cell suspension was prone to form a water-in-oil micro-droplets at the junction area of the T-shaped microchannel. The micro-droplet formed was soon sheared off from the aqueous stream because of the shear force of the cross flowing oil. Such a design has also been proved feasible to produce micro-droplets in several previous studies [33,40,41,42].

**Figure 2:**
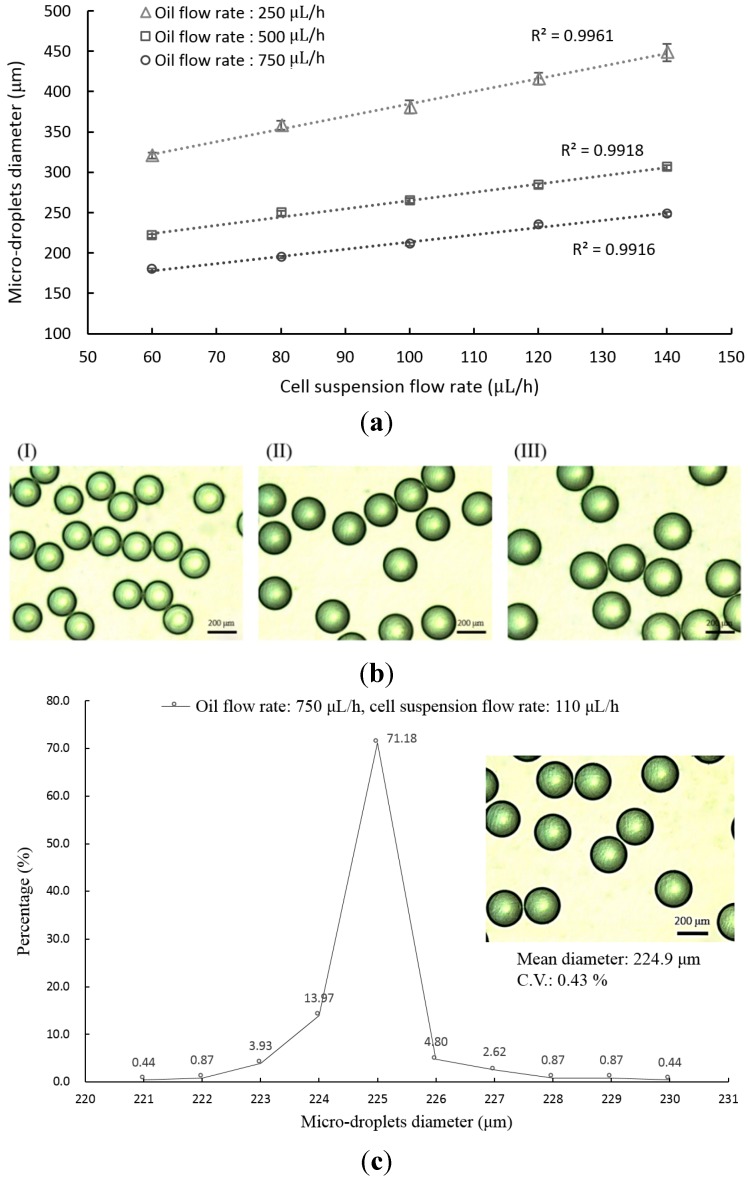

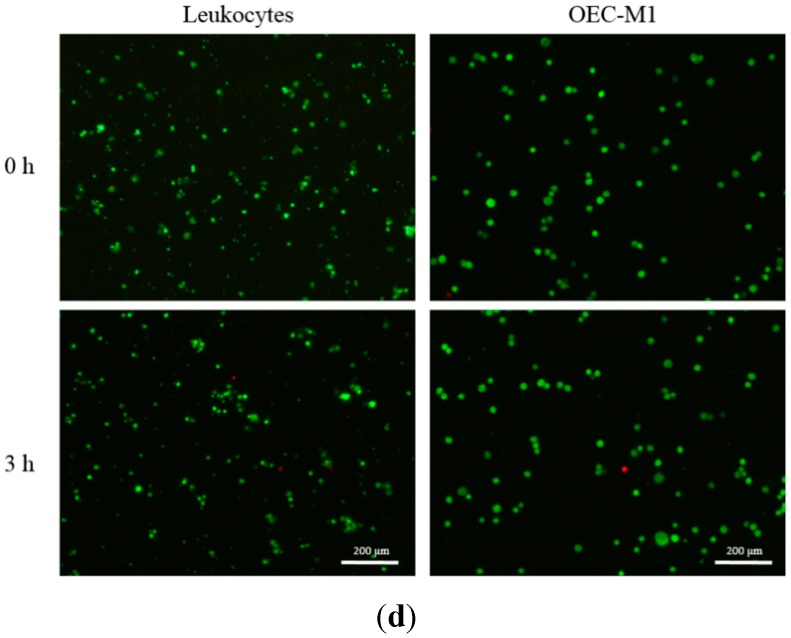
(**a**) The quantitative relationship between the flow rates of oil and cell suspension, and the resultant size (diameter) of micro-droplets; (**b**) Microscopic images of micro-droplets generated under three different operating conditions (oil flow rate: all 750 µL·h^−1^; cell suspension flow rate: (I) 60, (II) 100, and (III) 140 µL·h^−1^); (**c**) The size distribution of the micro-droplets (Oil flow rate: 750 µL·h^−1^, Cell suspension flow rate: 110 µL·h^−1^; Inset image: microscopic images of micro-droplet); (**d**) Microscopic observation of cell viability after micro-droplet-based microencapsulation process, and after 3 h static incubation using the Live/Dead^®^ fluorescent dye. Green and red dots represent live and dead cells, respectively. The left and right images show the leukocytes, and OEC-M1 cells, respectively.

Based on the abovementioned working mechanism, the input flow rates of oil, and cell suspension play an important role in determining the size of the micro-droplets generated. To find out the quantitative link between them, experiments were carried out. It is not unexpected that the size of micro-droplets decreased with the increase of oil flow rate under a given cell suspension flow rate (Figure 2a). At a given oil flow rate, results revealed that the diameter of the generated micro-droplets increased linearly (R^2^: 0.99) with the increase of cell suspension flow rate. Within the experimental conditions investigated, overall, micro-droplets with a diameter range of 179–248 µm can be generated in a size-controllable manner through the manipulation of the flow rates of oil and cell suspension. Figure 2b shows microscopic images of the micro-droplets generated under three different operating conditions (oil flow rate: all 750 µL·h^−1^; cell suspension flow rate: 60 (left), 100 (middle), and 140 (right) µL·h^−1^) with the corresponding average diameter of 179.4 ± 1.4, 210.8 ± 1.6 and 248.1 ± 2.3 µm, respectively. 

Based on our preliminary tests, the flow rates of oil and cell suspension were set at 750 and 110 µL·h^−1^, respectively, to produce the size-uniform micro-droplets with diameter of about 225 µm for the cell microencapsulation and the following optical detection of lactic acid. As mentioned earlier, the uniformity of the micro-droplets generated is an important technical issue in terms of the subsequent applications or investigations. In this case, the non-uniform cell-containing micro-droplets could complicate the subsequent optical detection of lactic acid. To examine the uniformity of the micro-droplets generated by the proposed microfluidic device, experiment was carried out. Figure 2c exhibited the size distribution of the micro-droplets generated by the set operating condition, in which the inset photograph showed the micro-droplets produced. The mean value, and C.V. of the micro-droplet diameter were then calculated to be 224.9 ± 1.0 µm, and 0.43%, respectively. It can be proved from the investigation that micro-droplets with uniform size can be produced through the proposed method. In addition to the size uniformity of micro-droplets, the viability of the cells contained in such micro-droplets is also a critical issue for cell microencapsulation. In a microfluidic system, the substance is normally transported through micro-level channels. Under such scale feature, the transported substance could be subject to high shear stress [43,44]. Such a physical environment could damage the cells, and further cause their death. As discussed earlier, this study mainly makes use of a metabolic feature of cancer cells to detect these cells. For an application of this kind, the cells encapsulated in the micro-droplets have to be kept viable. To ensure this, the cell viability of cancer cells encapsulated in these micro-droplets was examined. Figure 2d shows images of the OEC-M1 cells, and leukocytes soon after the microencapsulation process, and after 3 h of static incubation in the micro-droplets, in which the green and red dots represented the live and dead cells, respectively. For the two cell species explored, overall, the encapsulated cells maintained cell viabilities as high as 96 ± 2%, indicating that the proposed method and/or the operating conditions used were cell-friendly.

### 3.2. The Feasibility of Using the Proposed Microfluidic Optical Sensing Device for Live Cancer Cell Detection

**Figure 3:**
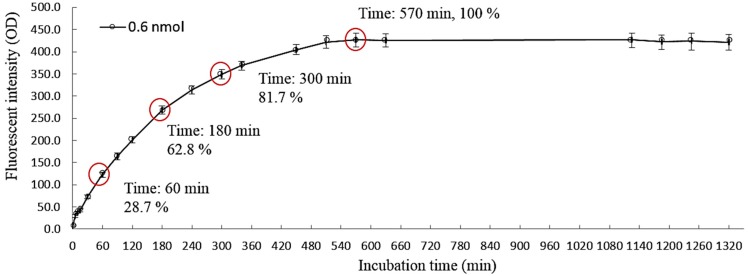
The observation of fluorescent intensity with time. The fluorescence-based lactate reagent was mixed with lactate solution. The fluorescence detection was carried out periodically for up to 22 h.

In this study, live cancer cells in micro-droplets were detected indirectly through their metabolic feature of lactic acid production. For the measurement of lactic acid in the micro-droplets, a fluorescent reagent was supplemented in the cell suspension tested before loading into the proposed microfluidic device. After the micro-droplet generation process (Figure 1b), the generated cell-containing micro-droplets were kept static in the incubation zone (Figure 1a) both for the cancer cells to produce lactic acid, and for the development of fluorescence. In an optical-based bio-sensing, the proper development of fluorescence is important in terms of the resultant sensing performances (e.g. sensitivity). In order to find out the incubation time, after which the fluorescence development is adequate for the following optical sensing, an experiment was carried out. Results (Figure 3) disclosed that the fluorescent intensity increased with the increase of incubation time, in which the fluorescent intensity reached the maximum level when the incubation time was higher than 570 min. Within the experimental conditions explored, moreover, such fluorescence maintained at a stable level for up to 1320 min (22 h). In this work, 180 min (3 h) incubation time was adopted, which is the compromise between the fluorescent intensity developed (62.8% of the maximum fluorescent intensity) and the overall assay time.

**Figure 4:**
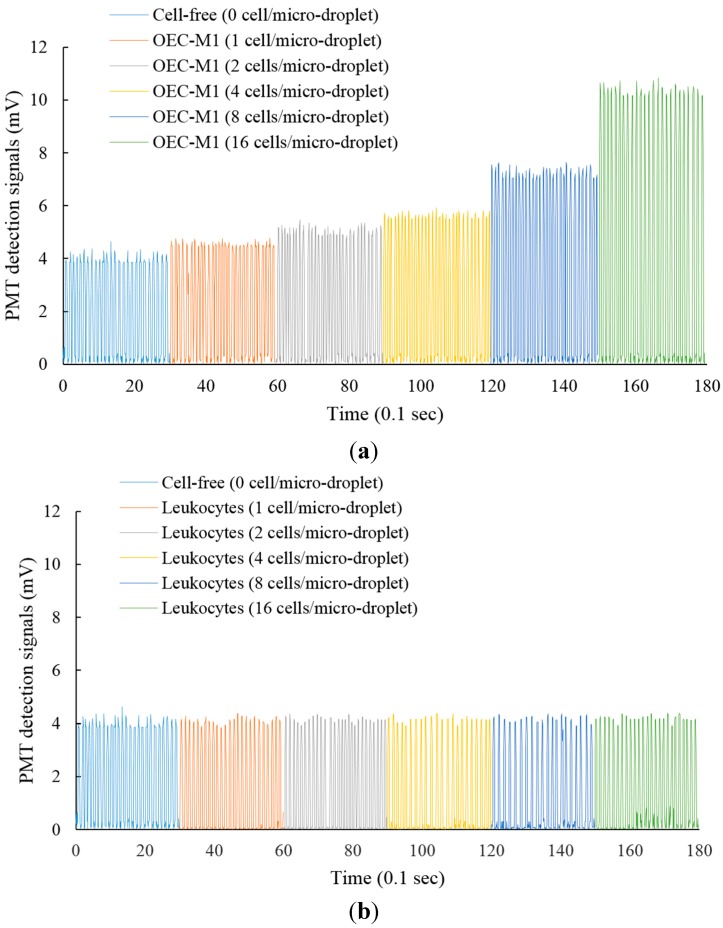

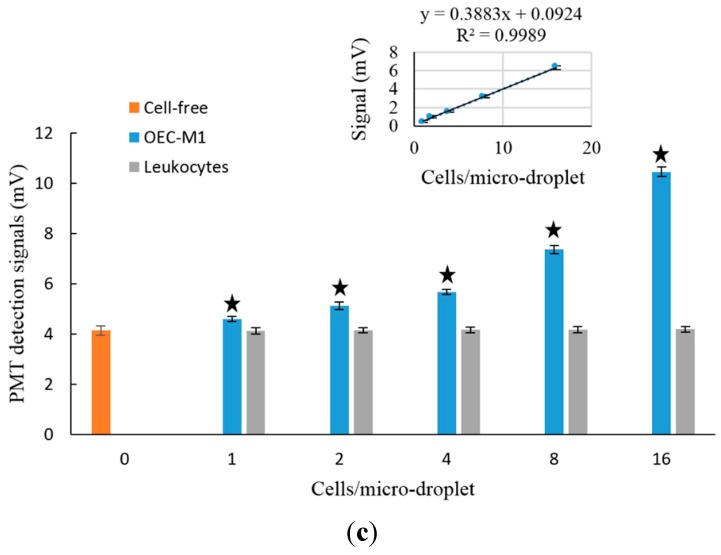
The detection signals of micro-droplets containing different levels (1, 2, 4, 8 and 16 cells/micro-droplet) of (**a**) OEC-M1 cells; (**b**) leukocytes; and (**c**) The quantitative relationship between the detection signals and the cell (OEC-M1 cells and leukocytes) number in each micro-droplet. The fluorescence detection was carried out after 3 h static incubation. The results are displayed as the mean ± the standard deviation of three separate experiments. Significant differences are expressed as ★ (*p* < 0.05).

**Figure 5:**
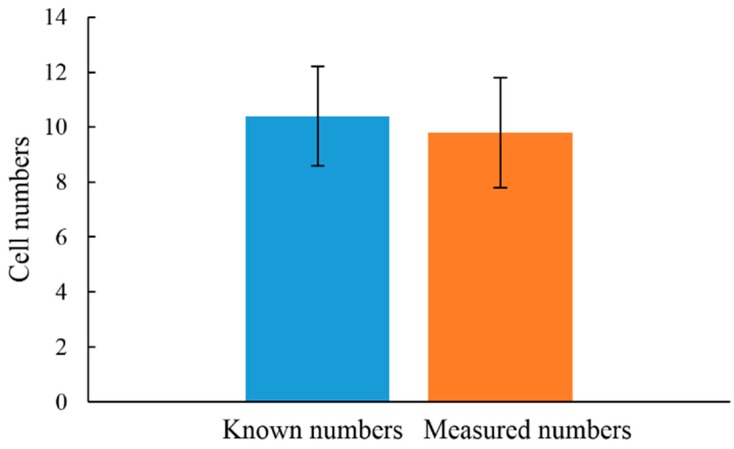
The experimental quantification of the number of OEC-M1 cells through the proposed method and its comparison with the calculated number. The results are displayed as the mean ± the standard deviation of 3 separate experiments.

In the conventional CTC isolation or detection methods, the erythrocytes are normally removed through different approaches [20], leaving behind the CTCs and the leukocytes in the sample. The CTCs are then isolated or identified from the leukocyte background. To further test the feasibility of using such approach for the quantitative evaluation of the live cancer cells in the abovementioned cell mixture, cell suspensions of leukocytes and OEC-M1 cells with different cell densities were loaded into the wells of 96-well microplate for a 3 h static incubation. After incubation, the cell suspensions were loaded into the microfluidic device for the micro-droplet generation and the following optical detection of lactic acid based on the established experimental setup (Figure 1d,e). In this study, the fluorescent optical signals were converted to electrical signals via the DAQ card. Figure 4a and b shows the detection signals (mV) of the micro-droplets containing different cell numbers of OEC-M1 cells, and leukocytes, respectively. In Figure 4a,b, each peak represents the detection signal of each micro-droplet. For quantitative comparison purpose, the average detection signals of the conditions investigated were evaluated and statistically analyzed as shown in Figure 4c. For the leukocyte case, the detection signals of the micro-droplets containing different levels of leukocytes, and of the cell-free micro-droplets showed no statistical difference (*p* > 0.05, ANOVA). Implying that under the experimental conditions explored, the detection signals were insensitive to the existence and the quantity of such cells in the micro-droplets. For the OEC-M1 cells, conversely, the detection signals were significantly influenced by the existence, as well as the cell number of such cells in the micro-droplets (*p* < 0.05, ANOVA). It can be clearly found that the detection signals increased linearly with the increase of the cell number in each micro-droplet (Figure 4c: Inset), indicating the lactic acid production was proportional to the number of encapsulated cancer cells. Based on above quantitative relationship, the cell number of cancer cells in a cell suspension can be determined indirectly by the lactic acid produced by the live cancer cells. After the above fundamental investigations, a known number of cancer cells was prepared in a cell suspension (OEC-M1 cells: 10 cells; volume: 10 µL), and was loaded into the microfluidic device. After the cell-containing micro-droplet generation and the in-chip static incubation processes as described earlier, the detection signals were then experimentally measured to quantify the number of cancer cells in such prepared cell suspension based on the quantitative relationship described in Figure 4c. The results (Figure 5) revealed that the measured cell numbers of cancer cells had no statistical difference (*p* > 0.05, ANOVA) with the cell numbers of cancer cells added into the cell suspension, indicating such method was capable of quantifying the live cancer cells in a cell suspension. Although the above proof-of-concept study has demonstrated the feasibility of using the proposed method for CTC detection there is one important issue which needs to be concerned in the practical application of such method. Because a blood sample will normally contain lactic acid it is necessary to isolate and separate the cells from the blood sample (e.g., via centrifugation) and then to suspend the cells in a cell culture medium for the CTC detection so as to avoid the interference caused by the lactic acid in the blood sample.

## 4. Conclusions

In this study, a microfluidic device to microencapsulate a cell suspension into multiple tiny water-in-oil micro-droplets (diameter: 225 µm) for lactic acid measurement was designed and fabricated. Results showed that the microfluidic device was able to generate the cell-containing micro-droplets in a size-tunable, size-uniform (C.V.: 0.43%), and cell-friendly (cell viability: 96%) manner. To justify the feasibility of using such approach to quantify cancer cells, the fluorescence signals of micro-droplets generated from the waste cell culture mediums with different cell numbers of leukocytes, and cultured human oral cancer (OEC-M1) cells were detected and compared. Results showed that the detection signals were proportional to the number of cancer cells within the micro-droplets (R^2^: 0.99), whereas such signals were insensitive to the existence and number of leukocytes within. This could indicate that such a method was capable of detecting the targeted cancer cells without the interference caused by the cell species. Furthermore, a known number of cancer cells in a prepared cell suspension was detected and then compared with the real number of such cells. Results revealed that there was no significant difference between them. Overall, this study has proposed a microfluidic-based optical sensing device to detect live CTCs in an effective and economical manner.
